# Detection of thermotolerant coliforms and SARS-CoV-2 RNA in sewage and recreational waters in the Ecuadorian coast: A call for improving water quality regulation

**DOI:** 10.1371/journal.pone.0302000

**Published:** 2024-05-06

**Authors:** Maritza Cárdenas-Calle, Leandro Patiño, Beatriz Pernia, Roberto Erazo, Carlos Muñoz, Magaly Valencia-Avellan, Mariana Lozada, Mary Regato-Arrata, Miguel Barrera, Segundo Aquino, Stefania Fuentes, Javier Duque, Luis Velázquez-Araque, Bertha Carpio, Carlos Méndez-Roman, Carlos Calle, Guillermo Cárdenas, David Guizado-Herrera, Clara Lucía Tello, Verónica Bravo-Basantes, Jhannelle Francis, Miguel Uyaguari

**Affiliations:** 1 Interinstitutional Network for the Study of Aquatic Ecosystems of Ecuador, Guayaquil, Guayas, Ecuador; 2 Ambiente Sociedad & Empresa Research Group, University of Guayaquil, Guayaquil, Guayas, Ecuador; 3 Faculty of Chemical Engineering, University of Guayaquil, Guayaquil, Guayas, Ecuador; 4 Fundación Bioelit, Guayaquil, Guayas, Ecuador; 5 National Institute for Public Health Research–INSPI- Dr. Leopoldo Izquieta Pérez, Technical Direction of Research, Development and Innovation, Guayaquil, Guayas, Ecuador; 6 Faculty of Natural Sciences, Natural Resources Research Institute, University of Guayaquil, Guayaquil, Guayas, Ecuador; 7 Labcestta, Guayaquil, Guayas, Ecuador; 8 Facultad del Mar y Medio Ambiente, Universidad del Pacífico, Guayaquil, Guayas, Ecuador; 9 Environmental Microbiology Laboratory, Institute of Biology of Marine Organisms, CONICET, Puerto Madryn, Chubut, Argentina; 10 National Institute for Public Health Research–INSPI- Dr. Leopoldo Izquieta Pérez, National Reference Center for Exanthematous, Gastroenteric and Vector-borne Viruses, Guayaquil, Guayas, Ecuador; 11 Dirección del Medio Ambiente, Gobierno Provincial de Santa Elena, Santa Elena, Ecuador; 12 Área Nacional de Recreación Playas Villamil, Ministerio de Ambiente Agua y Transición Ecológica, Playas, Ecuador; 13 Department of Microbiology, University of Manitoba, Winnipeg, Manitoba, Canada; Universidad San Francisco de Quito, ECUADOR

## Abstract

Wastewater surveillance represents an alternative approach to regulating contamination and the early detection of infectious agents and outbreaks of diseases of public health importance. This study evaluated domestic wastewater effects on recreational waters in estuarine and seawater bodies in Guayas and Santa Elena provinces in Ecuador, South America. Fecal indicator bacteria (thermotolerant coliforms) served as key indicators for evaluation. Physical, chemical, and microbiological quality markers following the Ecuadorian environmental quality standard and the discharge of effluents to the water resource were analyzed. Samples were collected from 44 coastal sites and 2 oxidation lagoons during the dry and rainy seasons of 2020 and 2021, respectively. SARS-CoV-2 RNA was detected in samples with higher *E. coli* concentrations using reverse transcription quantitative PCR to detect the genes N and ORF1ab. All samples analyzed for SARS-CoV-2 showed Ct ˂ 40 for at least one gene. Four samples showed at least 20 genome copies of gene N per reaction. These were at an artisanal fishing port, an estuarine area (Palmar), a recreational bay, and an oxidation lagoon. A moderate correlation was found between SARS-CoV-2 RNA, thermotolerant coliform and *E. coli* (p-value ≤ 0.0037), and a strong and positive correlation between thermotolerant coliform and *E. coli*. (p-value ≤ 0.00001), highlighting the utility of these established parameters as a proxy of the virus. Significant differences were found in the concentrations of thermotolerant coliforms between seasons (p-value = 0.016) and sites (p-value = 0.005). The highest levels of coliforms were found in the dry season (63000 MPN/100 mL) in Anconcito and during the rainy season (14000 MPN/100 mL) at Esterillo in Playas County. It is recommended that the decentralized autonomous governments of the surveyed provinces in Ecuador implement urgent corrective actions and establish medium-term mechanisms to minimize a potential contamination route. Additional parameters must be included in the monitoring, such as *Enterococcus* and intestinal parasites, due to their public health implications. In the oxidation lagoons, maintenance actions must be carried out, including the dissolution of sediments, an increase in water retention times, and in situ treatment of the sludge, to improve the system’s performance.

## Introduction

Improving sanitation infrastructure and enhancing water quality are key objectives within the United Nations’ sustainable development goals for 2030. According to the UNICEF-WHO, 2019, in Latin America and the Caribbean, only 38% of the population has access to a safe wastewater treatment service and another 50% has only access at a basic level. In this region, recreational water including rivers, lakes, and coastal waters are exposed to anthropogenic activities, which could deteriorate its quality [1–4]. Fecal contamination is one of the most common issues. This may occur through point source discharges (e.g. discharges of treated sewage/wastewater), runoff from urban or agricultural areas, and bather and animal excreta [5]. Besides the quality affectation of the ecosystem, water fecal contamination has been associated with risk to human health [6]. For example, surveillance data from the United States of America (USA) during 2009 and 2010, showed 24 recreational water disease outbreaks associated with the use of natural waters, 13 of which were attributed to human pathogens including *Campylobacter jejuni*, *E. coli* O157:H7, *Shigella sonnei*, *Giardia intestinalis*, *Cryptosporidium* spp., *Giardia intestinalis*, mammalian and avian schistosomes, and Norovirus.

Monitoring of recreational water for fecal contamination is a common policy in developed countries. It is conducted using certain bacterial species as indicators [7,8], which include total and thermotolerant coliforms. These bacteria are not postulated as the causative agents of illnesses in bathers but appear to behave similarly to the actual fecal-derived pathogens [9]. *E. coli*, in particular, has been proposed as one of the best indicators for gastroenteritis and dermal symptoms caused by seawater bathing [10], given its high frequency in domestic drains and longer survival time in seawater with respect to other coliforms. Other non-pathogenic fecal microorganisms such as intestinal enterococci are now being recommended to assess health risks in recreational waters [11]. Thermotolerant coliforms have been monitored on beaches to detect impacts from sewage [12]. Sewage sources on beaches include cesspools, septic tanks, and leaking sanitary sewer systems [13].

The Severe Acute Respiratory Syndrome Coronavirus 2 (SARS-CoV-2) causing the COVID-19 pandemic is also excreted in feces [14]. Its main route of transmission occurs via respiratory droplets and human contact [15] while the environmental air quality and surfaces contaminated by the virus is a factor that determines the speed of spread [16]. The virus nucleic acid has been detected in untreated and treated sewage in Australia, China, the USA, the Netherlands, France, Spain, and Italy but also in rivers impacted by urban wastewater in Japan and Ecuador [17,18]. Within this context, there are concerns that transmission from wastewater might occur or that viable viruses from its effluents reach water habitats [19]. However, based on indirect evidence, the risk of infection with SARS-CoV-2 from wastewater and recreational water bodies is considered low according to the Centers of Disease Control and Prevention [20,21], as virus exposure to environmental conditions as temperature, seasonal variations, and salinity would reduce its viability [22]. While the risk of transmission of SARS-CoV-2 from water bodies needs further evidence, there are growing recommendations for its surveillance as an early warning tool for predicting outbreaks, occurrence, prevalence, and potential public health risks in the communities [23,24].

Monitoring of fecal contamination in recreational waters is not usually enforced in developing countries. Despite this, recreational waters are widely used and leisure activities represent one of the main economic incomes for the local population. In tropical countries such as Ecuador, beaches are visited by tourists from all over the country and abroad throughout the year. The beaches are crowded especially on holidays, four of them (Christmas, New Year’s Eve, Carnival and Easter) occur during the rainy season of the Ecuadorian Coast (December-April) making these coastal areas susceptible to runoff events. Ecuador has a total of 421 wastewater treatment plants; of these, 129 are located in the coastal region [25], however, the effectiveness of their treatments and capacity for the growing population is poorly known. The purpose of this study was to assess water quality of these vulnerable coastal areas, including the presence of SARS-CoV-2, and infer the possible correlation of water quality-associated variables with the detection of the virus. We analyzed the most visited beaches in Ecuador and two oxidation lagoons at Guayas and Santa Elena Provinces during restrictions (dry season in 2020) and reopening of beaches (rainy season in 2021).

## Materials and methods

Water quality parameters were selected according to the criteria present in the Ministerial Agreement 097A to prevent and control water pollution in Ecuador. We measured parameters for two quality criteria: i) admissible quality for the preservation of aquatic life and wildlife in freshwater, marine and estuarine waters, II) water quality for recreational waters by primary and secondary contact [26]. All the parameters enforced by the Ecuadorian regulation are shown in supplementary material (S1 Table). In addition, we monitored SARS-CoV-2 RNA by RT-PCR.

### Study sites

**Fig 1:**
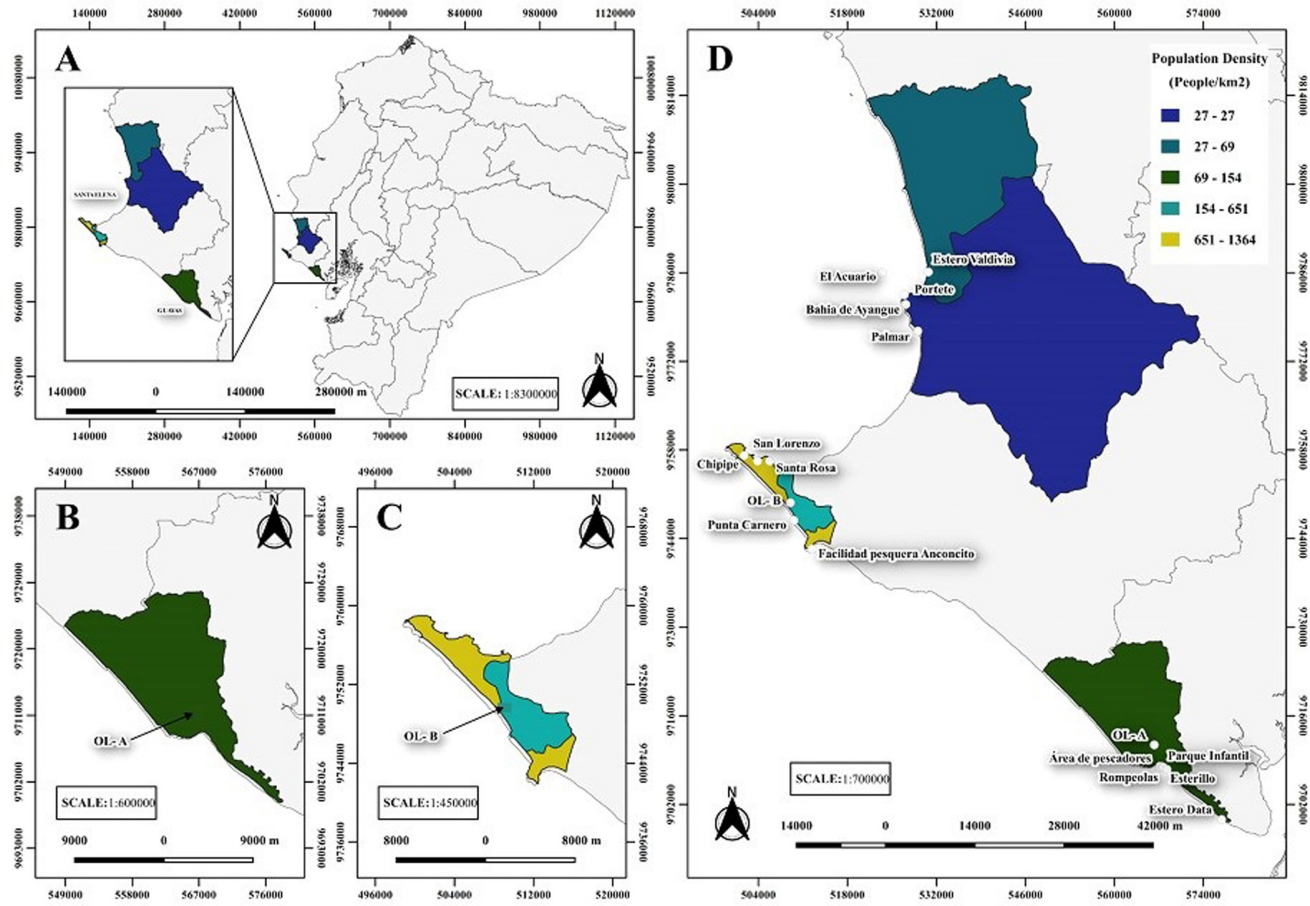
Sampling sites locations and population density of studied sites. (A) Sample site location within Guayas and Santa Elena Provinces in Ecuador. (B) Oxidation lagoons of Playas (OL-A) situated in Guayas province. (C) Oxidation lagoons of Punta Carnero (OL-B) situated in Santa Elena province. (D) The sites studied and its population density.

The study area was located between latitude 1° 56’ 5.8" S and 2° 43’ 24.4" S, and longitude 80° 43’ 24.6" W and 80° 18’ 18.5" W along the Ecuadorian coast between Guayas and Santa Elena Provinces (Fig 1, Table 1). Seventeen sampling sites were selected: fifteen were coastal areas including artisanal fishing ports, estuaries, recreational beaches and bays, and a diving area located 10 km from the coast (coded A to O); two were oxidation lagoons (Ols), one from Playas-Guayas Province (OL-A) and one from Punta Carnero-Santa Elena Province (OL-B). All areas were studied during the Ecuadorian confinement established to reduce the impact of the COVID-19 pandemic during the dry season in July 2020 (coded A1-O2) and after the confinement in the rainy season in January 2021 (coded A2-O2). The corresponding sampling permits were obtained from the Ministry of Environment of Ecuador.

**Table 1:** Sampling sites. Provinces, locations, site code, site description, and coordinates from study sites.

Province	Site Code	Replicate	Station	Site description	Coordinates	
					Latitude	Longitude
**Guayas**	A	1-2-3	Rompeolas	Beach (next to hotel)	2° 38’ 37,983" S	80° 23’ 49,495" W
	B	1-2-3	Área de Pescadores	Artisanal Fishing Processing area	2° 38’ 29,188" S	80° 23’ 44,835" W
	C	1-2-3	Parque Infantil	Beach	2° 38’ 34,715" S	80° 23’ 26,147" W
	D	1-2-3	Esterillo	Water discharge channel from the city and oxidation lagoon	2° 38’ 45,584" S	80° 23’ 8,071" W
	E	1-2-3	Estero Data	Estuarine area influenced by shrimp farms and human settlements	2° 43’ 24,368" S	80° 18’ 18,493" W
	OL-A	1-2-3	OL-A: HidroPlayas	Oxidation lagoon	2° 36’ 38,536" S	80° 24’ 13,063" W
**Santa Elena**	F	1-2-3	Facilidad Pesquera Anconcito	Artisanal Fishing Processing area	2° 19’ 52,367" S	80° 53’ 17,239" W
	G	1-2-3	Punta Carnero 3	Estuarine area used for recreational activities	2° 13’ 29,231" S	80° 58’ 15,877" W
	H	1-2-3	Chipipe 1	Beach (next to hotel and human settlements)	2° 11’ 50,382" S	80° 59’ 2,597" W
	I	1-2-3	San Lorenzo 1	Beach (next to hotel and human settlements)	2° 12’ 19,433" S	80° 57’ 54,284" W
	J	1-2-3	Santa Rosa 1	Artisanal Fishing Processing area	2° 12’ 19,627" S	80° 56’ 52,866" W
	K	1-2-3	Palmar	Estuarine area, fishing boats landing port influenced by human settlements	2° 1’ 19,98" S	80° 43’ 55,92" W
	L	1-2-3	Bahia de Ayangue 1 Derecho	Bay, recreational area and boat landing area	1° 58’ 52,44" S	80° 45’ 19,5" W
	M	1-2-3	Portete Grande Casa del Sol	Beach influenced by shrimp´s laboratory and pipes human settlement	1° 58’ 9,9" S	80° 45’ 16,8" W
	N	1-2-3	El Acuario	Open water located near El Pelado Islote used for recreational scuba diving	1° 56’ 10,92" S	80° 47’ 21" W
	O	1–2	Estero Valdivia 1	Estuarine area influenced by pig farm and human settlements	1° 56’ 5,81" S	80° 43’ 24,6" W
	OL-B	1-2-3	OL-B: Punta Carnero	Oxidation lagoon	2° 15’ 29,504" S	80° 55’ 5,581" W

The cities of General Villamil Playas (Guayas Province) and Salinas (Santa Elena Province) have 41,935 and 68,675 inhabitants, respectively [27]. The main economic activities in both cities are fishing, gastronomic, and beach tourism that take place throughout the year. These places receive national tourists from the months of December to April (coastal region holiday season); and from August to October (highland region holiday season). Santa Elena has a sanitary system that covers 60% of the urban area. This sanitary network collects wastewater to a pumping station and then towards oxidation lagoons, where they also receive influents from Salinas, Santa Rosa, Muey and La Libertad. Wastewater receives a secondary treatment within oxidation lagoons that involves disinfection with chlorine before treated effluents are discharged into the sea, while Playas has a facultative lagoon [28].

### Physical-chemical parameters

Fourteen variables were measured during the sampling period. Four parameters were obtained *in situ* at each sampling site: Temperature (Temp), pH, salinity (Sal), and dissolved oxygen (DO), using YSI HQ30d multiparameter probes. Seven parameters were measured in the laboratory: chemical oxygen demand (COD), biological oxygen demand (BOD), oils & fats (OF), surfactants or tensoactives (TENS), total residual chlorine (Cl), ammonium (NH4), total suspended solids (TSS), according to standard methods [29]. For the laboratory analyses, triplicate 1L water samples were collected during the low tide using a telescopic rod sampler at a depth of 30 cm, and collected into sterilized plastic bottles. Samples were stored at 4°C in the dark, transported to the laboratory and processed within 24 h of collection.

COD analysis was performed by VIS spectrophotometry at a wavelength of 620 nm using a HACH DR/6000 spectrophotometer. The sample was previously digested for 2 hours at 150°C with sulfuric acid 18.1M and a strong oxidizing agent (potassium dichromate 0.04 M). The BOD analysis was carried out by calculating the difference between the initial oxygen and the final oxygen determined in the sample after 5 days of incubation with a standard microbial community [29]. Surfactant analysis was performed by extraction in benzene from an acidic aqueous medium containing excess methylene blue, followed by countercurrent washing with water, and the determination of the blue color by spectrophotometry at 652 nm using the HACH DR/spectrophotometer 6000. The analysis of OF was carried out by gravimetry by extraction with hexane [29]. TSS determination was performed by gravimetry after filtering the sample using a 0.45 μm pore diameter filter, drying the residue on the filter and determining the weight gain. The analysis of residual free Chlorine was carried out by spectrophotometry using the HACH DR/6000 spectrophotometer, after the addition of the indicator N, N-diethyl-p-phenylenediamine (DPD). The NH4 analysis was carried out following APHA (2017), a mineral stabilizer and the dispersing agent are applied to the sample that helps the formation of the reaction of the Nessler reagent with N-ammoniacal ions, forming the yellow color which is measured by spectrophotometry.

### Microbiological parameters

The microbiological parameters included thermotolerant coliforms (TC) and *Escherichia coli* bacterial numbers (EC). The analysis of TC was performed using the multiple tube fermentation technique, estimating the bacterial density using the most probable number (MPN) method [29]. EC analysis was performed using the membrane filtration technique using the selective growth medium m-ColiBlue24 and incubating the samples at 44.5°C [29]. Simultaneous detection of total coliforms and *E. coli* by Dual-Chromogen (m-ColiBlue24) membrane filter procedure was conducted. The volumes processed were the following: thermotolerant coliforms by the multiple tube fermentation technique (MPN): 10 mL, 0.1 mL, 0.01 mL in some cases and in other cases, 0.1 mL, 0.01 mL, 0.001 mL depending on the sample. *E. coli* by the membrane filter technique (CFU): 10 mL in some cases and 1 mL in others as indicated in the Standard Methods for Examination of Water and Wastewater Ed 23 section 9222B, 4a. Densities in both cases were determined by the Most Probable Number (MPN) method [30].

### SARS-CoV-2 analysis

The analysis of SAR-CoV-2 was conducted at the Multidisciplinary Center for Research of the National Institute for Public Health Research of Ecuador (INSPI). Water samples were processed into a biosafety cabinet Class II, Type A 2. One of the key factors for virus detection in water is the use of methods that allow the concentration of viral particles in enough amounts to be detected by the diagnostic test of interest. Here we used a method for concentration of organic particles based on viral adsorption–precipitation which has shown to be effective for SARS-CoV-2 concentration, using 40 mL of each sample [31,32]. All supernatants from the different stages of the viral concentration procedure were treated with chlorine overnight prior to its elimination through the laboratory’s piping. RNA was extracted using commercial QIAamp Viral RNA Mini Kit (Qiagen Sciences, Germantown, MD, USA) following the manufacturer’s instructions. The survey of SARS-CoV-2 RNA was performed using the 2019-nCoV Nucleic Acid Diagnostic Kit (Sansure Biotech), which targets the N and ORF1ab viral genes, and includes a positive control for SARS-CoV-2. The RT-PCR assay was set up as described by the manufacturer including positive and negative template controls. Reactions were run on a BIO-RAD CFX96 Real-Time PCR System, with the following thermocycling conditions: one cycle of 50°C for 30 seconds, one cycle if 95°C for 2 minutes, and 45 cycles of 95°C for 15 seconds and 60°C for 1 minute. For the interpretation of results, we adopted the kit recommendations for clinical samples: a RT-PCR amplification with cycle threshold (Ct) values <40 for any or both genes N and ORF1 ab was considered positive. Standard quantification curves were obtained for the N gene using the commercially available 2019-nCoV N positive control (IDT), a plasmid containing the complete nucleocapsid gene of SARS-CoV-2, provided at 2 x 10^5^ copies/μL. We used 10-fold serial dilutions from 2 x 10^4^ to 2 x 10 copies/μL. Each dilution was submitted to RT-PCR in triplicate under the conditions explained above, using 1 μl of template. The lowest concentration at which all 3 replicates were positive was treated as the limit of detection (LoD). SARS-CoV-2 RNA detected in samples was quantified as gene copy numbers (GCNs) per reaction by plotting the quantification cycles (Ct) to the standard curve.

### Statistical analysis

Correlation between variables was visualized using the draftsman plot. Kolmogorov-Smirnov test was conducted for the assessment of normality of data distribution, and the Levene test for homoscedasticity [33]. Subsequently, given the lack of normality for the whole data set, a non-parametric test (Kruskal-Wallis) was performed [34] to identify statistical differences between sampling sites regarding water physicochemical and microbiological parameters including Ct values <40 for N1 and N2 genes.

The entire sampling seasons were considered to calculate maximum, minimum and mean values of physicochemical parameters. Multivariate analysis of all sampled sites was carried out based on physicochemical and microbiological parameters [35] only for the data collected in beaches. The Euclidean distance was used to construct a similarity matrix from the fourth-root transformed data for each replicate sample at each site in each site and season. The matrix was then subjected to Non-Metric Multidimensional Scaling (n-MDS) ordination previous individual transformation Log (V+0.1) of the variables TC, EC, TENS and TSS. Two-way crossed ANOSIM tests were used to identify significant differences between seasons and sites. In each test, the null hypothesis that no significant differences are found between season and sites was rejected if the significance level was p≤0.001. The R statistic value was used to ascertain the extent of any significant differences [36]. Multivariate analyses were performed using the statistical package Primer-E [36], version 7.0.20. Finally, the distribution of TC in the coastal areas and OL and pollutant critical points were plotted on the Ecuadorian map using QGIS program version 3.4.3 and Spearman’s correlation was calculated to evaluate the association between environmental variables and SARS-CoV-2 performed in the programming language R and Rstudio (version 4.3.0).

### Compliance with water Ecuadorian environmental regulations

The maximum permitted levels of the studied variables were compared with tables 2, 6 and 10 of Book VI from the Unified Text of Secondary Legislation of the Ministry of the Environment (TULSMA): Environmental quality standard and effluent discharges to water bodies, Ministerial Agreement 097A [26].

## Results

### Physical-chemical parameters

**Fig 2:**
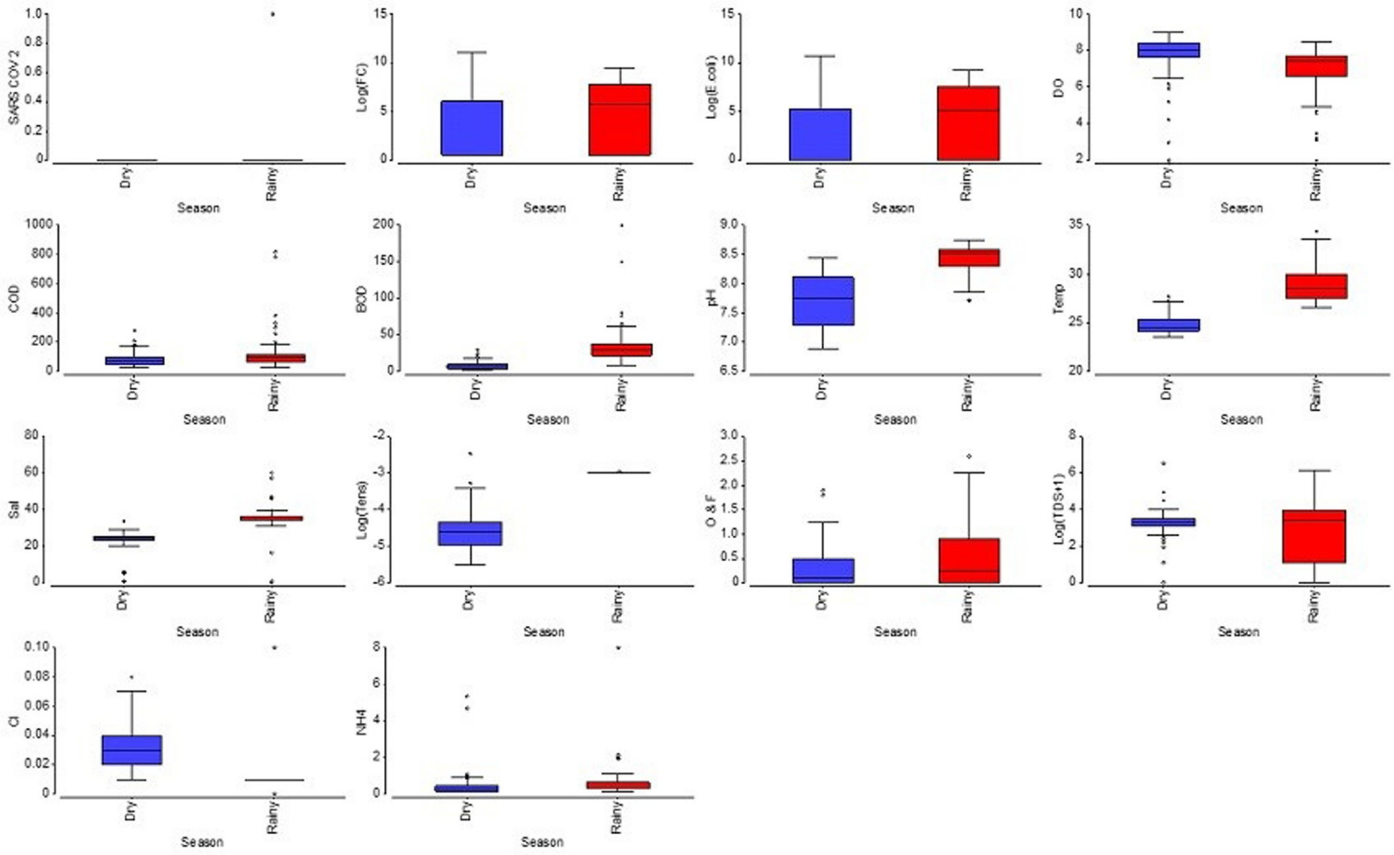
Physico-chemical and biological quality parameters assessed in seawater of beaches during dry (blue) and rainy (red) seasons. TC: Thermotolerant coliforms; DO (mg/L): Dissolved oxygen; COD (mg/L): Chemical oxygen demand; BOD (mg/L): Biological oxygen demand; temp (Celsius): Temperature; Sal (Practical salinity units): Salinity; tens (mg/L): Tensoactives; O & F (mg/L): Oils and fats; TDS (mg/L): Total dissolved solids; Cl (mg/L): Chlorine; NH_4_ (mg/L): Ammonium.

Maximum, minimum and mean values obtained from physical and chemical parameters at each sampling site for dry season (2020) and rainy season (2021) are shown in S1 Table. The temperature ranged from 23.6°C to 34.4°C with higher values in the rainy season (January) and lowest in the dry season (July), due to differences between the season period and time of survey (early in the morning or past midday). A higher temperature was observed in Estero Valdivia and Esterillo. The higher TSS values were observed in three locations: Ayangue in the Estero Valdivia (698 mg/L) in an area close to a pig farm, Area de Pescadores (114 mg/L) and Esterillo (86 mg/L) located near restaurants in Playas, mainly in the rainy season (S1 Table). Dissolved oxygen concentration fluctuated between 2 and 9 mg/L, with higher concentration in the rainy season (July) and lowest concentration associated with the estuarine area called Estero Valdivia and Area de Pescadores. Concentrations lower than 5 mg/L (below the recommended concentration for the preservation of the flora and fauna) were found at fishing facilities (Anconcito), estuaries (Esterillo and Valdivia) and fishing ports (Area de Pescadores). COD ranged from 27 mg/L to 820 mg/L. The highest values were registered at the outer and middle branches of Estero Valdivia during the rainy season; these values exceeded the permissible COD limits for the preservation of flora and fauna according to environmental laws in Ecuador (400 mg/L). Other sites with high levels although within limits were found at the estuarine zones such as: Estero Data 1 (384 mg/L), Estero Data 3 (302 mg/L), Palmar 3 (260 mg/L) and Punta Carnero (210 mg/L) (Fig 2). BOD ranged from 2 to 200 mg/L, higher levels were observed only at Estero Valdivia in the middle (150 mg/L) and outer estuary (200 mg/L) during the rainy season (Fig 2).

The pH ranged from 6.88 to 8.75, the lowest values were recorded in July of 2020 at Estero Data (6.88) and in the rain season (7.72) at Esterillo. Salinity ranged from 0.5 to 60.2 PSU; an alteration in salinity was observed in intertidal zones due to the influence of wastewater from Playas county, and in landing and commercialization of fishing in Area de Pescadores; estuarine area (Esterillo). The highest salinity values were found in the external area from Estero Valdivia where high temperatures increase evaporation rate, increasing salinity in the area near the mouth of the sea during the rainy season. Tensoactives showed values between 0.004–0.085 mg/L, the highest concentrations were observed in Punta Carnero 1 during February 2021 (S1 Table). In addition, NH4 ranged from 0.12 to 8 mg/L with highest levels at intertidal zones where domestic wastewater is discharged (Area de Pescadores), and estuarine zones such as Esterillo and Estero Valdivia (S1 Table). The concentrations of residual free chlorine and surfactants were found below the detection limit (10 mg/L and 0.05 mg/L, respectively). There were only significant (p <0.05) differences between sampling sites for Temp, Sal and OF. In this context, OF ranged from 0.07 to 2.6 mg/L, exhibiting the highest values in the rainy season (January 2021). OF values tended to be higher (≥ 2 mg/L) in fishing boats and landing ports where the artisanal fishing fleet is sheltered (Anconcito, Portete Grande, Area de Pescadores and Estero). This increase coincided with the beaches that were reopened with higher tourist activity. Similar trends were observed in water channels with wastewater and industrial runoff coming from Playas´ downtown discharged in the intertidal zone at Esterillo. A two-way crossed ANOSIM demonstrated that water quality was influenced by both site and season (R = -0.76; p = 0.001).

### Microbiological assessment

**Fig 3:**
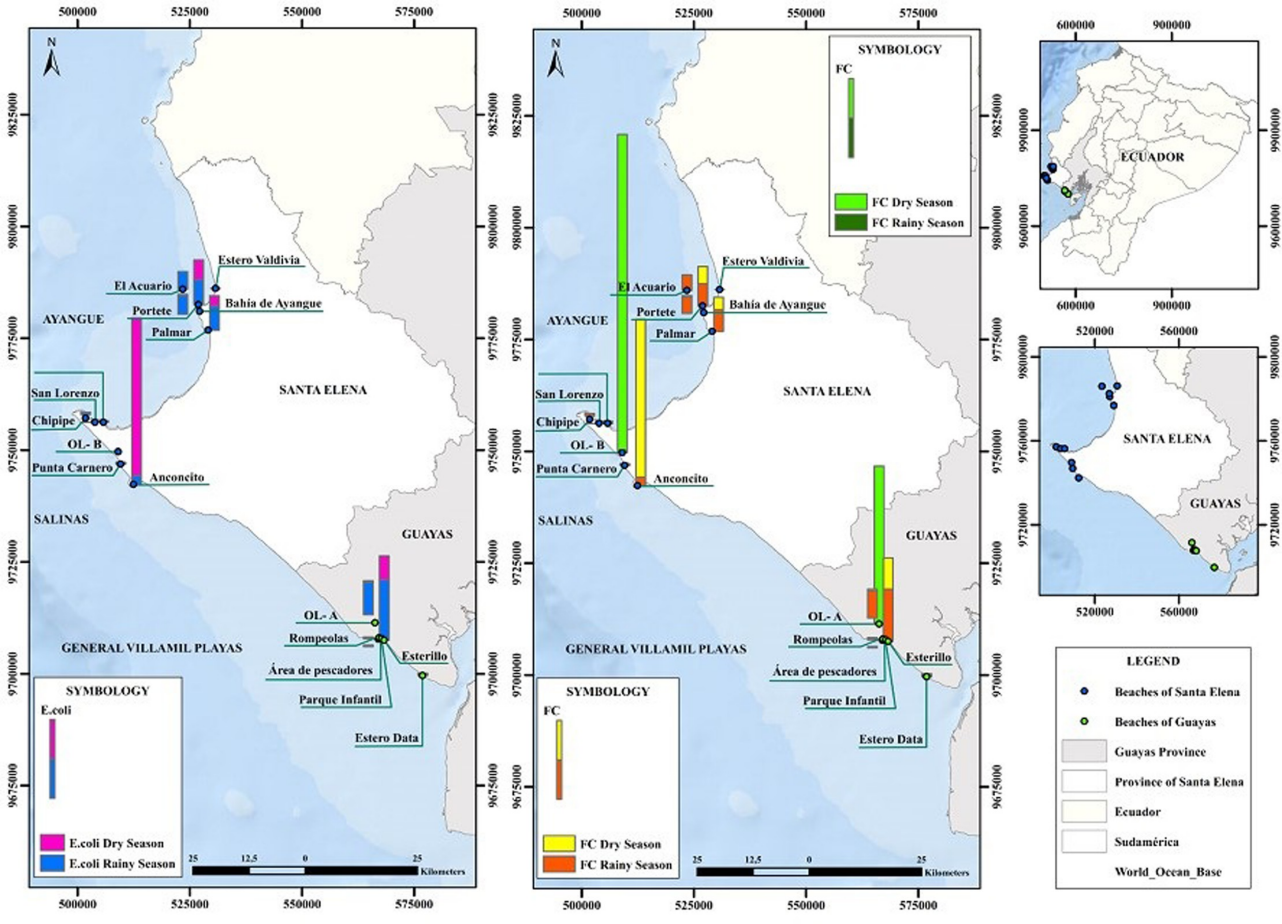
Spatial distribution of *E. coli* (left) and thermotolerant coliforms (right) in the seawater and wastewater treatment systems during 2020–2021. The concentration of thermotolerant coliforms in OL-A and OL-B are light green in dry season and dark green in the rainy season, Thermotolerant coliforms on beaches are yellow in the dry season and orange in the rainy season.

TC counts were very variable, ranging from 1.8 to 63,000 MPN/100 mL. The highest levels were found in samples collected at artisanal fishing ports, estuaries, and urbanizations. Although the highest value was found during the dry season at Anconcito Artisanal Fishing Port, in general, the higher levels and number of sites were observed in the rainy season (Fig 3). Both TC and EC were consistently higher in seawater and wastewater discharged in estuarine zones compared to shoreline sand. Values were high at a fish landing and management facility located at Anconcito Artesanal Fishing Port, at a channel of domestic wastewater (Esterillo 1 and 2), in areas next to restaurants and public toilets, on the coastline near urbanizations (Portete Grande Casa del Sol), and in an estuarine area influenced by urban settlement (Palmar 1) (Fig 3). The highest TC values were detected in Anconcito (63000 MPN/100 mL), Esterillo (9,200 MPN/100 mL), Portete (7000 MPN/100 mL) and Palmar (4000 MPN/100 mL). The same trends were registered for EC (Fig 3).

There was a statistically significant difference between sites for TC (p = 0.03) and EC (p = 0.03). Also, both TC and EC were higher (p<0.05 and p = 0.01, respectively) in the rainy season when beaches opened for tourism compared to the dry season and closed beaches. TC were highest in Santa Elena in relation to Playas (p<0.05 and p = 0.01) in both survey times. However, a higher number of sites showed water polluted with TC and *E. coli* in the area from Ayangue, which is a preferred area by tourists due to its calm waters, especially in Ayangue Bay and surroundings. In fact, only ten of the surveyed sites could be suitable for recreational purposes according to environmental laws in Ecuador through ministerial agreement 097A [37]. The recommended limit to TC is 200 MPN/100 mL according to the current environmental regulations for water in Ecuador (TULSMA). These sites were Rompeolas, Parque Infantil 2 and Estero Data, Punta Carnero 1, Punta Carnero 2, Chipipe 1, Chipipe 2; San Lorenzo 1,2,3 and Estero Valdivia.

### Relation between seawater water quality and environmental factors

**Fig 4:**
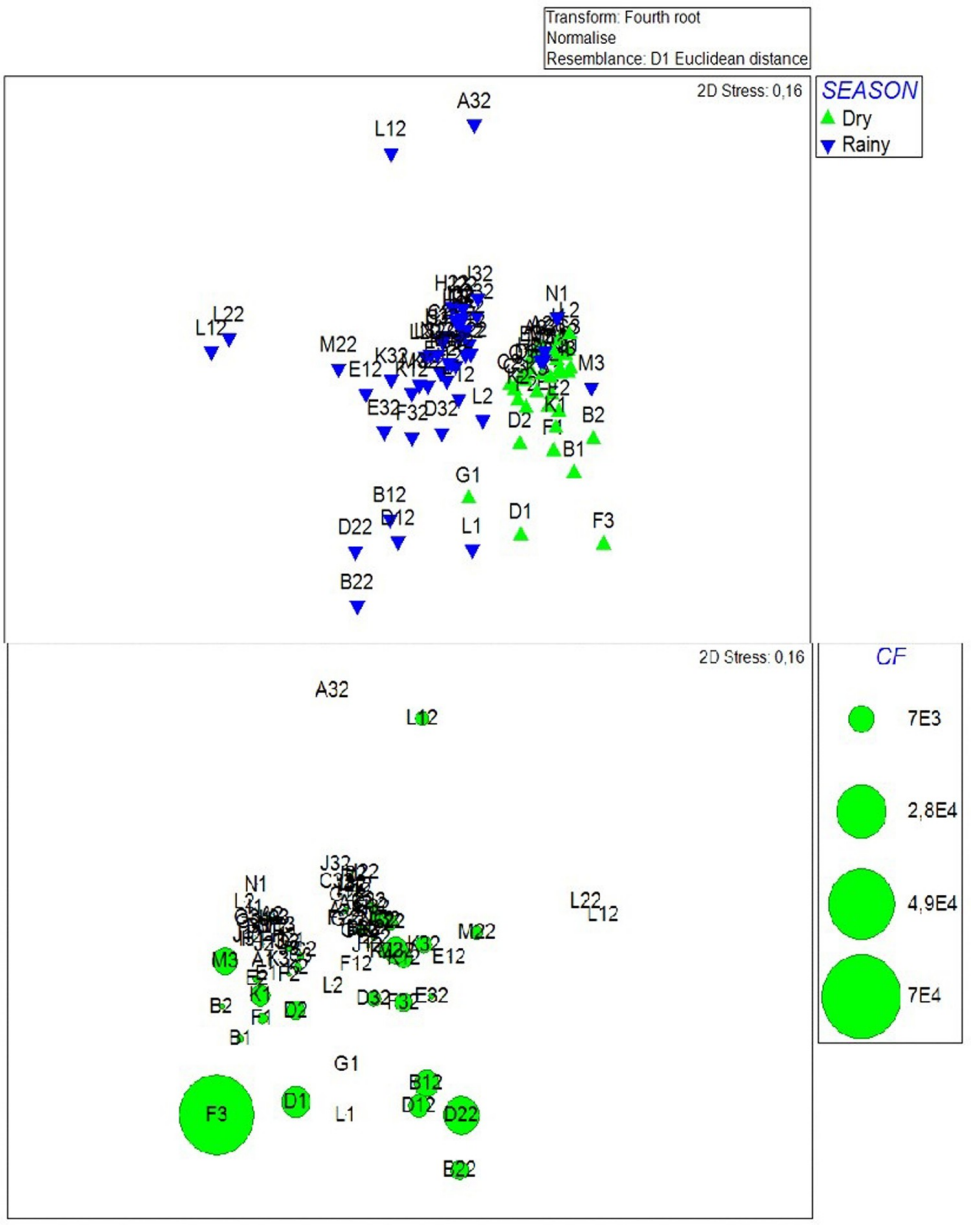
Non-metric multidimensional scaling ordination, derived from Euclidean distance constructed using square root transformed total chemical, physical and microbiological parameters at 42 sampling sites during dry season (2020) and rainy season (2021). The upper plot has used for all variables and seasons and is coded for season, while the lower plot was restricted to Thermotolerant Coliforms for a seasons.

The non-metric multidimensional scaling ordination showed that there were differences in water quality according to seasonality (dry vs rainy seasons) (Fig 4). The similarity of studied sites was influenced by the levels of TENS, Temp, pH, BOD, Sal, Cl, EC, and DO. The SIMPER routine showed the variables with most contribution to differences were TENS (10.54%); Temp (9.94%); pH (8.87%), BOD (7.46%) and Sal (7.15%).

### Microbiological pollution in wastewater oxidation lagoons

The main microbiological, physical and chemical parameters in OLs are shown in Table 2. The concentration of TC fluctuated between 1.8 to 9.2 x 10^6^ MPN/100mL. The highest levels were recorded in Santa Elena during 2020, with maximum levels registered in the entrance of Punta Carnero (OL-B, 9.2 x 10^6^ MPN/100 mL) while lower concentrations were recorded in the Playas´ lagoons (1.2 x 10^5^ MPN/100 mL). The levels observed in the output of both oxidation lagoons were high, with values above the allowed limit (2000 MPN/100 mL) for discharge from wastewater to seawater in Ecuador (Table 2). The high levels of TC recorded at the output of the oxidation ponds show low performance and low efficiency in the removal of organic matter and microorganisms in these systems.

**Table 2:** Microbiological and physicochemical parameters of oxidation lagoons at Guayas (Playas) and Santa Elena (Punta Carnero).

Variables	Input OLA Playas	Output OLA Playas	Input OLA Playas	Output OLA Playas	Input OLB Punta Carnero	Output OLB Punta Carnero	Input OLB Punta Carnero	Output OLB Punta Carnero	Maximum limit Levels of Environmental Parameter in Marine Waters in Ecuador	W Test-p value	
**TC** (MPN/100mL)	120000	110000	53000	1.8	9200000	700000	24000	6300	2000	W = 0	P = 0.0303826
**COD** (mg/L)	684	337	566	258	533	222	634	249	400	W = 8.0	P = 0.885229
**BOD5** (mg/L)	320	160	260	120	270	110	310	110	200	W = 5.5	P = 0.56136
**pH**	7.48	7.66	7.54	7.7	7.6	7.74	7.56	7.8	6–9	W = 9.0	P = 0.885229
**Temperature** (°C)	17.1	17.3	23.2	21.4	24.8	24.7	24.1	23.7	<35	W = 8.0	P = 0.885229
**Tensoactives** (mg/L)	0.19	0.06	2.92	**0.05**	1.2	3.36	2.5	0.052	0.5	W = 6.0	P = 0.665002
**Oils & fats** (mg/L)	5.8	3.2	9.2	5.3	3.1	2.2	28.3	2.3	30	W = 12.0	P = 0.31232
**Turbidity**	142	90.5	76.1	76.1	159	57.4	62.7	60	NA	W = 4.0	P = 0.309423
**Color**	110	50.26	32.84	25.18	60.46	36.17	35.52	26.12	NA	W = 0	P = 0.0303826
**Survey date**	1/9/2020	1/9/2020	25/2/2021	25/2/2021	4/9/2020	4/9/2020	26/2/2021	26/2/2021			

### SARS-CoV-2 analysis

The standard curve from the N gene of SARS-CoV-2 showed a linear dynamic range with a R2 value of 0.997, slope -3.196, and *y* intercept 38.435. The LoD as per the lowest concentration at which all 3 replicates for the standard curve were positive was 20 GCNs per reaction with a Ct value of 34.45 ± 0.97. The results of the RT-PCR are shown in Table 3. All samples analysed for SARS-CoV-2 showed Ct ˂ 40 for at least one gene. Four samples the GCNs per reaction were under the LoD established for this assay for the gene and comprised an artisanal fishing port (Facilidad Pesquera Anconcito), an estuarine area (Palmar), and a recreational bay (Portete Grande Casa del Sol), which were positive during the dry season, and an oxidation lagoon from Playas-Guayas Province that was positive during the rainy season. The remaining samples showed amplification signals over the LoD of the gene N: both genes N and ORF1ab were detected from both oxidation lagoons sampled during the dry season. For the results of other sampling sites by season see Table 3.

**Table 3:** Ct detected for genes N and ORF1ab by reverse transcription-polymerase chain reaction in two oxidation lagoons and five coastal waters exceeding permissible limits of TC.

Season	Sample Code	Sample site	N Ct	ORF1ab Ct	Interpretation
**Dry**	OL-A	Oxidation Lagoon Playas	36.80	38.72	Positive
**Dry**	OL-B	Oxidation Lagoon Santa Elena	36.44	38.99	Positive
**Dry**	B	Area de Pescadores	36.98	> 40	Positive
**Dry**	F	Facilidad Pesquera Anconcito	**35.41**	> 40	Positive
**Dry**	K	Palmar	**33.21**	> 40	Positive
**Dry**	M	Portete Grande Casa del Sol	**34.41**	> 40	Positive
**Rainy**	L	Bahía de Ayangue	39.75	38.73	Positive
**Rainy**	D	Estero Data	38.54	40	Positive
**Rainy**	OL-A	Oxidation Lagoon Playas	**35.16**	40	Positive
**Rainy**	OL-B	Oxidation Lagoon Santa Elena	40	38.46	Positive

## Discussion

Ecuador beaches are a popular destination for recreation and touristic activities, and are therefore heavily populated all year long, with peaks during the dry season (July). In this study we showed that 9 out of 15 beaches (60%) have biological contamination with thermotolerant coliforms and *E. coli*. This pollution is normally associated with wastewater treatment, runoff, contaminated water from rain or subsoil by seepage, river mouths bathers and animal shedding into seawater [38]. It is important to mention that this research was conducted during COVID-19 pandemic, before a vaccine was available, and while the use of beaches was prohibited. Anconcito and Esterillo were the beaches with the highest pollution in terms of thermotolerant coliforms. A possible explanation for these highly contaminated beaches may be associated with higher thermotolerant coliform counts and high BOD and COD observed in the raw sewage and oxidation ponds of these locations.

We observed that the oxidation lagoons were discharging very high concentrations of coliforms in their effluents, an indication of the inefficiency in the removal of this type of microorganisms. The low water retention times in the lagoons, caused by a significant increase of water in the population caused by their stay in their homes during the pandemic could be further increasing this discharge, significantly contaminating the beaches and estuaries. Considering that discharges of low-quality treated, or even untreated water (such as from homes not connected to the public sewerage network, discharges without outfalls directly into estuaries and beaches) and considering their mixing by waves and currents, it can be said that contamination is permanently fed by household discharges that can even move from the coastline to open waters. This has been observed in El Esterillo, Data de Posorja, Palmar and Ayangue. In this last area for example, the dispersion plume reached 5.30 km along the coastline from Portete Grande Casa del Sol to El Pelado Islet, where fecal coliform concentrations reached 7000 MPN/100 mL and 2100 MPN/100mL respectively in the rainy season. These values are much higher in relation to the ones determined in 2013 in El Pelado Islet (31 NMP/100mL, [39]). This pattern has been observed in other developing countries as well, such as Colombia [40] and other countries from Latin America and the Caribbean.

This study also revealed that the abundance of coliforms was higher at the inlet and outlet of oxidation ponds during the beach closure season compared to the beach opening season when confinement measures were lifted, suggesting that the source may be the overloading of the ponds due to confinement. The oxidation ponds are highly efficient when operating under appropriate conditions of residence time and organic load. However, the coliform data in the studied oxidation ponds suggest that an evaluation and improvement of the methods used to eliminate bacteria and viruses in these systems is required. Even though the levels of coliforms were very high in the oxidation ponds, they were lower on the beaches where these waters were eventually discharged, and the presence of the SARS-CoV-2 RNA was not detectable, probably due to the dilution effect in the sea and to salinity conditions, incidence of ultraviolet radiation and the temperature of seawater [41,42].

Another impact of the discharge of inefficiently treated wastewater to the beaches was evidenced by the low levels of oxygen found (less than 5 mg/L). Due to the organic load of wastewater, effluent discharged from wastewater treatment facilities often contributes to the level of oxygen demand of the receiving water, due to the greater depletion of dissolved oxygen in the surface waters that receive water with poorly treated residuals [43]. For example, DO levels in effluents from various wastewater treatment facilities in South Africa were typically lower than the required standard of 8 to 10 mg/L, and this condition was shown to negatively affect the aquatic ecosystem [1]. In our work, high levels of COD were observed in the beaches near urbanizations, fishing facilities, estuaries, and bays, whilst the highest levels of BOD were found in the Estero Valdivia, showing there is organic contamination in water. Therefore, there could be negative impacts of the lagoon systems in Playas and Santa Elena, impacting the local flora and fauna of these sites, in agreement with previous studies carried out in Playas and Santa Elena [28].

Finding of SARS-CoV-2 RNA at the oxidation lagoons sampled was expected as the virus is excreted in feces, but positive RT-PCR results in artisanal fishing ports and natural sampling sites confirm fecal contamination is reaching the surrounding natural ecosystem. Nevertheless, RNA detection does not imply virus viability, for SARS-CoV-2 persistence of infectious particles in water matrices is temperature dependent [44]. In tropical countries, it could also be affected by other environmental variables including seasonal seawater temperature, exposure to UV radiation, and salinity, among others. A laboratory assay using the coronavirus murine hepatitis virus spiked-in raw wastewater has shown a 90% reduction of infectivity at 25°C after 13 h but longer infectivity rate at 10°C after 36 h. Using the same assays, the SARS-CoV-1, genetically closer to SARS-CoV-2, has been found to be viable for up to 14 days at 4°C and 2–3 days at ∼20°C in raw sewage [45,46]. In fact, SARS-CoV-2 was found to be infectious after 2.3 days at 20°C and 3.8 days at 4°C in river water, and 1.1 days at 20°C and 2.2 days at 4°C in seawater [44]. The rapid decay of SARS-CoV-2 in environmental water supports the suggestion of the CDC that viral transmission through contact with contaminated waters is low. This result, however, shows that human-derived microorganisms could reach the coastal environments, highlighting the possibility that oro-fecal transmited and antimicrobial resistant pathogens with longer persistence rates could be introduced to recreational water [11,47,48]. SARS-CoV-2 RNA was moderately correlated with TC and *E. coli* abundance. This remarks the potential of using wastewater for monitoring human pathogens excreted in feces [49].

Developing countries such as Ecuador have weak regulations for water quality, and surveillance of recreational water is not in place. The use of recreational waters promotes one of the main economic activities in Ecuador, hence, survey of its quality to guarantee users and fauna health should become a priority. Our results emphasize the need for secondary or biological treatment such as activated sludge or moving bed bioreactors, rather than primary/physical treatment as conducted in these areas with oxidation ponds. The longer period of time for retention in wastewater treatment plants could help inactivation of the coronavirus and dissappearance of other pathogens, potentiated with an increase in temperature [50,51].

Moreover, there is a need to develop a monitoring program of water quality that incorporates other indicators of fecal contamination beyond parameters analyzed in this study that are part of the parameters required by the current environmental regulations in Ecuador to assess fecal contamination in coastal marine ecosystems. Enterococci species that are recommended as bacterial fecal indicators and thermo-tolerant coliforms as *Klebsiella* spp., *Enterobacter* spp. and *Citrobacter* spp. [52] for estuarine and marine waters as opportunistic pathogens in humans that have evolved a number of mechanisms for resistance gene transfer and have the potential to transfer their resistance genes to other more pathogenic gram-positive bacteria, an education program for owners of hotels and restaurants at tourist beaches, regarding good practices in food manufacturing and prevention of foodborne diseases (ETA).

## Conclusions

In this work, water quality of seawater and wastewater discharges in two regions of Ecuador were analyzed during two seasons of the COVID pandemic. Notably, a great proportion of beaches exceeded the maximum limit allowed in water for recreational activities according to Ecuadorian environmental legislation. SARS-CoV-2 RNA was present in seawater and outfalls of estuaries, highlighting the need to improve sanitation in these ecosystems. Its abundance was moderately correlated with other microbiological indicators, namely thermotolerant coliforms and *E. coli*. The implementation of a water quality monitoring program that includes physical, chemical, and microbiological parameters to guarantee the water quality for recreational and productive activities is urgent.

Strengthening the capacities of technicians from decentralized Autonomous Governments to monitor contamination in estuaries, bays, wetlands, tourist areas with hotel infrastructure, fishing facilities, human settlements and shrimp farms, is greatly needed to prevent waterborne diseases. These policies will have positive impacts in public health, health of aquatic ecosystems and economic activities.

## Supporting information

S1 Table Physico-chemical parameters in seawater of the beaches under study. (PDF)

S1 Fig Heat map showing Spearman’s rank correlation analysis between physico-chemical and biological parameters collected during sampling events. Euclidean distance was used as dissimilarity measure between parameters.
(TIF)
