# Supplementary material for: Detection of thermotolerant coliforms and SARS-CoV-2 RNA in sewage and recreational waters in the Ecuadorian coast: A call for improving water quality regulation
Source: PLoS One. 2024 May 6;19(5):e0302000. doi: 10.1371/journal.pone.0302000 (PMC11073733; doi:10.1371/journal.pone.0302000)
Supplement: S1 Table — (PDF) [file pone.0302000.s001.pdf]

Stations	Station	Season	Temperature (°C)	TSS (mg/L)	pH	Salinity (UPS)	DO (mg/L)	BOD (mg/L)	COD (mg/L)	O&F (mg/L)	NH_4 (mg/L)	TENS (mg/L)	Cl (mg/L)
**Rompeolas**	A	Dry	24.20 (24.10-24.30)	24.00 (23.00-32.00)	7.98 (7.47-8.04)	22.93 (21.90-23.53)	8.12 (8.04-8.14)	5.00 (2.00-7.00)	**80.00** (60.00-170.00)	0.00 (0.00-0.10)	**0.45** (0.25-0.60)	<0.05	0.02 (0.01-0.05)
		Rainy	30.00* (28.80-30.50)	6.00 (4.00-8.00)	**8.53*** (8.22-8.57)	**34.30*** (34.20-34.30)	7.35 (7.33-7.86)	17.00* (10.00-18.00)	**52.00** (30.00-56.00)	0.00 (0.00-0.00)	**0.30** (0.21-0.37)	<0.05	0.01 (0.01-0.01)
**Área de pescadores**	B	Dry	24.70 (24.50-25.30)	12.00 (2.00-26.00)	7.54 (7.20-7.56)	1.17 (0.72-23.36)	5.94 (4.23-8.13)	10.00 (5.00-15.00)	30.00 (27.00-50.00)	**0.20** (0.00-0.30)	**0.37** (0.29-0.40)	<0.05	0.01 (0.01-0.03)
		Rainy	**27.30*** (27.10-29.80)	84.00 (2.00-114.00)	**8.37*** (8.06-8.56)	0.90 (0.50-34.50)	5.83 (2.00-7.47)	44.00* (21.00-46.00)	**95.00*** (64.00-96.00)	**1.80** (0.30-2.00)	**1.99** (0.34-8.00)	<0.05	0.01 (0.01-0.01)
**Parque Infantil**	C	Dry	25.70 (25.60-26.00)	26.00 (22.00-28.00)	7.92 (7.82-8.09)	23.57 (23.33-23.60)	7.79 (7.72-7.92)	15.00 (12.00-20.00)	**130.00** (120.00-180.00)	0.00 (0.00-0.00)	**0.92** (0.89-0.98)	<0.05	0.07 (0.06-0.08)
		Rainy	**30.00*** (29.60-30.20)	4.00 (4.00-6.00)	**8.66*** (8.30-8.69)	34.30* (34.30-34.80)	7.45* (7.42-7.55)	32.00 (12.00-35.00)	**100.00** (40.00-104.00)	0.00 (0.00-0.00)	**0.38** (0.24-0.39)	<0.05	0.01 (0.01-0.01)
**Esterillo**	D	Dry	25.70	32.00	8.29	5.80	9.00	7.00	**80.00**	**0.30**	**1.06**	<0.05	0.03

Stations	Station	Season	Temperature (°C)	TSS (mg/L)	pH	Salinity (UPS)	DO (mg/L)	BOD (mg/L)	COD (mg/L)	O&F (mg/L)	NH_4 (mg/L)	TENS (mg/L)	Cl (mg/L)
			(25.10-26.20)	(22.00-36.00)	(7.68-8.45)	(5.47-23.37)	(7.84-9.00)	(5.00-10.00)	(60.00-100.00)	(0.10-1.80)	(0.25-4.70)		(0.02-0.04)
		Rainy	**31.60***	50.00	8.07	0.90	**3.47**	11.00	**50.00**	**1.10**	**1.93**	<0.05	0.01
			(31.40-33.40)	(42.00-86.00)	(7.72-8.42)	(0.80-16.50)	(3.10-5.20)	(8.4-42.00)	(34.00-89.00)	(0.50-1.70)	(0.16-2.14)		(0.01-0.01)
**Estero Data**	E	Dry	26.60	24.00	7.10	22.43	7.67	8.00	**90.00**	**1.00**	0.15	<0.05	**0.04**
			(25.40-27.20)	(14.00-34.00)	(6.88-8.36)	(21.28-27.16)	(7.07-9.00)	(6.00-8.00)	(70.00-90.00)	(0.10-1.90)	(0.12-0.19)		**(0.03-0.04)**
		Rainy	31.30*	38.00	7.92	34.30*	5.70*	**76.00**	**302.00**	**1.00**	**0.78**	<0.05	0.10*
			(31.00-31.30)	(4.00-52.00)	(7.73-8.29)	(21.28-27.16)	(4.59-7.05)	(32.00-80.00)	(96.00-384.00)	(0.07-2.00)	(0.36-0.98)		(0.01-0.10)
**Facilidad Pesquera Anconcito**	F	Dry	24.00	10.00	7.28	25.85	5.21	8.00	**90.00**	**0.50**	0.14	<0.05	**0.03**
			(23.90-24.10)	(8.00-14.00)	(7.20-7.30)	(25.40-25.94)	(2.98-6.17)	(6.00-18.00)	(70.00-160.00)	(0.50-0.60)	(0.13-0.16)		(0.03-0.070)
		Rainy	27.00*	42.00	**8.53***	36.00*	6.92*	**30.00**	**92.00**	**2.00**	**0.35**	<0.05	0.10
			(26.70-27.60)	(6.00-52.00)	(7.93-8.64)	(35.90-36.20)	(3.21-7.05)	(25.00-38.00)	(76.00-116.00)	(0.40-2.60)	(0.31-0.41)		(0.01-0.10)
**Punta Carnero**	G	Dry	24.50	26.00	7.30	24.99	8.16	5.00	**60.00**	0.00	0.17	<0.05	**0.02**
			(24.40-24.70)	(22.00-28.00)	(7.17-7.62)			(4.00-30.00)	(84.00-96.00)		(0.15-5.35)		

Stations	Station	Season	Temperature (°C)	TSS (mg/L)	pH	Salinity (UPS)	DO (mg/L)	BOD (mg/L)	COD (mg/L)	O&F (mg/L)	NH_4 (mg/L)	TENS (mg/L)	Cl (mg/L)
						(20.14-25.36)	(7.58-8.16)			(0.00-0.70)			(0.01-0.08)
		Rainy	26.90*<br>(26.60-27.30)	2.00<br>(2.00-6.00)	**8.48***<br>(8.47-8.67)	36.50*<br>(35.90-36.70)	7.80<br>(7.62-7.85)	**29.00**<br>(28.00-32.00)	**88.00**<br>(84.00-96.00)	**0.40**<br>(0.00-0.40)	**0.34**<br>(0.32-0.38)	<0.05	0.01<br>(0.01-0.01)
**Chipipe**	H	Dry	24.70<br>(24.50-24.70)	24.00<br>(22.00-26.00)	7.26<br>(7.00-7.86)	25.25<br>(24.96-25.26)	8.03<br>(7.84-8.04)	8.00<br>(7.00-10.00)	**90.00**<br>(80.00-100)	**0.10**<br>(0.10-0.10)	0.13<br>(0.13-0.19)	<0.05	**0.03**<br>(0.02-0.03)
		Rainy	28.40*<br>(27.90-28.50)	0.00<br>(0.00-0.00)	**8.59***<br>(8.34-8.75)	36.00<br>(36.00-36.10)	7.55*<br>(7.55-7.64)	**32.00***<br>(30.00-34.00)	**96.00***<br>(88.00-100.00)	**0.10**<br>(0.00-0.10)	**0.36**<br>(0.33-0.41)	<0.05	0.01<br>(0.01-0.01)
**San Lorenzo**	I	Dry	24.40<br>(24.20-27.70)	28.00<br>(26.00-30.00)	7.40<br>(7.15-7.46)	25.55<br>(25.22-25.76)	7.83<br>(7.70-8.45)	4.00<br>(3.00-4.00)	**50.00**<br>(40.00-50.00)	**0.10**<br>(0.00-0.20)	0.14<br>(0.12-0.15)	<0.05	**0.02**<br>(0.01-0.02)
		Rainy	27.60<br>(27.30-28.00)	0.00<br>(0.00-0.00)	**8.69***<br>**(8.68-8.73)**	36.00*<br>(36.00-36.10)	7.61*<br>(7.44-7.64)	**25.00***<br>(22.00-28.00)	**76.00***<br>(68.00-88.00)	**0.10**<br>(0.10-0.30)	**0.31**<br>(0.28-0.34)	<0.05	0.01<br>(0.01-0.01)
**Santa Rosa**	J	Dry	24.50<br>(24.40-24.60)	26.00<br>(22.00-28.00)	7.32<br>(7.25-8.29)	25.60	8.91	8.00<br>(3.00-.00)	**90.00**<br>(40.00-90.00)	**0.30**	0.18<br>(0.16-0.20)	<0.05	**0.03**

Stations	Station	Season	Temperature (°C)	TSS (mg/L)	pH	Salinity (UPS)	DO (mg/L)	BOD (mg/L)	COD (mg/L)	O&F (mg/L)	NH_4 (mg/L)	TENS (mg/L)	Cl (mg/L)
						(25.33-25.90)	(8.11-9.00)			(0.20-0.50)			(0.01-0.03)
		Rainy	28.80*<br>(27.40-29.00)	0.00<br>(0.00-24.00)	**8.66***<br>**(8.57-8.74)**	36.10*<br>(36.10-36.20)	7.75<br>(4.70-7.90)	18.00*<br>(16.00-21.00)	**56.00**<br>(48.00-64.00)	**0.00**<br>(0.00-1.20)	0.18<br>(0.15-0.25)	<0.05	0.01<br>(0.01-0.01)
**Palmar**	K	Dry	23.90<br>(23.60-24.00)	44.00<br>(6.00-90.00)	7.84<br>(7.67-7.92)	27.76<br>(27.41-33.29)	7.59<br>(6.68-9.00)	12.00<br>(4.00-15.00)	**120.00**<br>(50.00-130.00)	**0.70**<br>**(0.60-0.90)**	**0.33**<br>(0.28-0.61)	<0.05	**0.03**<br>(0.02-0.04)
		Rainy	28.20*<br>(27.80-28.40)	52.00<br>(42.00-52.00)	**8.54***<br>**(8.32-8.56)**	39.50*<br>(36.70-46.00)	7.30<br>(6.70-8.50)	**50.00**<br>(32.00-65.00)	**200.00**<br>(128.00-260.00)	**0.60**<br>**(0.50-0.90)**	**0.62**<br>(0.40-0.81)	<0.05	0.01<br>(0.01-0.01)
**Bahia de Ayangue**	L	Dry	24.30<br>(24.16-24.60)	30.00<br>(26.00-32.00)	8.10<br>(7.99-8.12)	25.12<br>(24.74-25.15)	8.29<br>(8.19-8.58)	4.00<br>(3.00-4.00)	**50.00**<br>(40.00-50.00)	0.00<br>(0.00-0.00)	0.22<br>(0.17-0.23)	<0.05	**0.02**<br>(0.01-0.02)
		Rainy	28.70*<br>(28.28-29.40)	52.00<br>(25.00-52.00)	**8.58**<br>**(8.50-8.59)**	36.40*<br>(36.30-36.40)	7.70<br>(7.60-7.80)	**30.00***<br>(28.00-34.00)	**116.00***<br>(108.00-136.00)	0.00<br>(0.00-0.00)	**0.36**<br>(0.34-0.42)	<0.05	0.01<br>(0.01-0.01)
**Portete**	M	Dry	24.30<br>(28.10-29.20)	34.00<br>(30.00-38.00)	8.16<br>(8.11-8.17)	24.69	8.74	5.00<br>(4.00-6.00)	**60.00**<br>(50.00-70.00)	**0.10**	**0.26**<br>(0.25-0.38)	<0.05	**0.02**

Stations	Station	Season	Temperature (°C)	TSS (mg/L)	pH	Salinity (UPS)	DO (mg/L)	BOD (mg/L)	COD (mg/L)	O&F (mg/L)	NH_4 (mg/L)	TENS (mg/L)	Cl (mg/L)
						(24.60-24.85)	(8.10-9.00)			**(0.00-0.10)**			(0.02-0.02)
		Rainy	28.20*<br>(28.10-29.20)	52.00<br>(52.00-52.00)	**8.49**<br>**(8.06-8.53)**	36.20*<br>(32.70-36.30)	7.90<br>(7.60-8.50)	**26.00***<br>(24.00-50.00)	**104.00**<br>(100.00-332.00)	**0.10**<br>**(0.10-2.00)**	**0.33**<br>(0.32-0.86)	<0.05	0.01<br>(0.01-0.01)
**El Acuario**	N	Dry	24.20<br>(24.20-24.20)	0.00<br>(0.00-0.00)	8.16<br>(8.16-8.16)	23.74<br>(23.74-23.74)	8.99<br>(8.99-8.99)	6.00<br>(6.00-6.00)	**70.00**<br>(70.00-70.00)	0.00<br>(0.00-0.00)	0.21<br>(0.021-0.021)	<0.05	**0.03**<br>(0.03-0.03)
		Rainy	28.60<br>(28.60-28.60)	52.00<br>(52.00-52.00)	**8.44**<br>(8.44-8.44)	36.20<br>(36.20-36.20)	8.50<br>(8.50-8.50)	**22.00**<br>(22.00-22.00)	**92.00**<br>(92.00-92.00)	0.00<br>(0.00-0.00)	**0.30**<br>(0.030-0.030)	<0.05	0.01<br>(0.01-0.01)
**Estero Valdivia**	O	Dry	26.29<br>(26.20-26.39)	**420.00**<br>**(142.00-698.00)**	8.17<br>(8.13-8.22)	34.05<br>(34.02-34.08)	**4.83**<br>**(2.00-7.65)**	**21.00**<br>(18.00-24.00)	**232.50**<br>(185.00-280.00)	**0.65**<br>**(0.50-0.80)**	**0.60**<br>(0.55-0.65)	<0.05	**0.06**<br>(0.05-0.07)
		Rainy	33.20*<br>(32.00-34.40)	**310.00**<br>**(160.00-460.00)**	8.24<br>(8.14-8.35)	**58.70***<br>(57.20-60.20)	6.60<br>(6.60-6.60)	**175.00***<br>(150.00-200.00)	**802.50***<br>(785.00-820.00)	**0.75**<br>**(0.60-0.90)**	**2.03**<br>(2.03-2.03)	<0.05	0.00<br>(0.00-0.00)
**MPL (Recreational Water Quality Criteria)**					6.5-8.3		5.84-6.64			Absence		0.5	

Stations	Station	Season	Temperature (°C)	TSS (mg/L)	pH	Salinity (UPS)	DO (mg/L)	BOD (mg/L)	COD (mg/L)	O&F (mg/L)	NH_4 (mg/L)	TENS (mg/L)	Cl (mg/L)
**MPL (Protection for aquatic life)**					6.5-9.5		5.84-6.64	20	40	0.3	0.25	0.5	0.01
**Kruskall-Wallis**			78.73	63.68	66.55	75.12	58.75	69.60	59.14	55.88	58.49		53.48
**P**			<0.001	<0.001	<0.001	<0.001	0.001	<0.001	0.001	0.002	0.001		0.004

*Indicates that there are significant differences between season in the same station according to U Mann-Whitney (P>0.05), MPL: maximum permissible level according to AM 097A. OD based on the criterion of minimum percentage of oxygen saturation 80% where the optimal values would be 7.3-8.3 OD mg / L at 25-30 ° C and 80% saturation = 5.84-6.64 mg / L.
