# Supplementary figures and images for: Detection of thermotolerant coliforms and SARS-CoV-2 RNA in sewage and recreational waters in the Ecuadorian coast: A call for improving water quality regulation
Source: PLoS One. 2024 May 6;19(5):e0302000. doi: 10.1371/journal.pone.0302000 (PMC11073733; doi:10.1371/journal.pone.0302000)

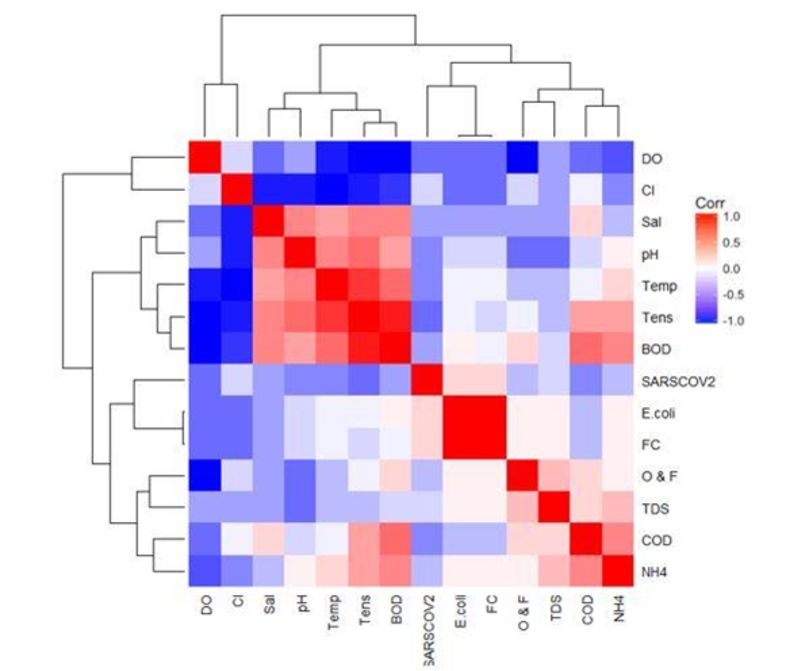

Supplement: S1 Fig — Euclidean distance was used as dissimilarity measure between parameters. (TIF) [file pone.0302000.s002.tif]
